# The longitudinal relationship between loneliness and problematic social networking site use in college students: the mediating role of trait- and state-fear of missing out

**DOI:** 10.3389/fpsyg.2025.1477239

**Published:** 2025-03-19

**Authors:** Yuhua Wang, Yufei Sun, Taiping Li

**Affiliations:** ^1^School of Education and Psychology, Hubei Engineering University, Xiaogan, China; ^2^School of Psychology, Beijing Sport University, Beijing, China; ^3^School of Education, Huazhong University of Science and Technology, Wuhan, China

**Keywords:** problematic social networking site use, loneliness, trait-fear of missing out, state-fear of missing out, multiple mediating effects

## Abstract

**Aims:**

This longitudinal study explored the mechanisms of loneliness, trait-fear of missing out (trait-FoMO), and state-fear of missing out (state-FoMO) on problematic social networking site use (PSNSU) among Chinese college students.

**Methods:**

Data were collected in two waves. Overall, 417 college students (45.08% male and 54.92% female, with an average age of 19.87 ± 1.05) completed measures of loneliness and PSNSU at the first time point (T1), and measures of trait-FoMO, state-FoMO, and PSNSU 12 months later (T2).

**Results:**

(1) There were significant positive correlations among loneliness, trait-FoMO, state-FoMO, and PSNSU; (2) Trait-FoMO and state-FoMO fully mediated the relationship between loneliness and PSNSU; (3) there were two paths of loneliness that influenced PSNSU: loneliness was associated with PSNSU through the mediating role of trait-FoMO alone and the chain mediating role of trait- and state-FoMO.

**Conclusion:**

This study highlights the need to accurately distinguish between trait-FoMO and state-FoMO, considering their different underlying mechanisms in addressing problematic network problems among college students.

## Introduction

Mobile social apps, such as WeChat, Weibo, QQ, and Xiaohongshu, have gradually become a new method of social communication, especially among college students in China. A national survey of 5,118 Chinese college students reported that 99.39% of them used social media every day, and 74.69% spent more than 4 h a day using social media (Chen et al., 2022). However, the relationship between social networking site use (SNSU) and individuals’ mental health is still controversial. Vuorre and Przybylski (2024) conducted a study in which data were collected from 2.4 million people aged 15 to 89 across 168 countries over the past 20 years. They found that social networking site use was not significantly associated with negative psychological outcomes. Additionally, the study found that the association between social networking site use and life satisfaction is more negative during specific periods of an individual’s adolescence than at other stages. Moreover, Kelly and Sharot (2024) found that the relationship between browsing more negative online information and poorer mental health is causal and bidirectional, creating a loop that may perpetuate mental health problems. Moreover, the researchers developed an intervention that altered web-browsing patterns to improve mood. Together, these studies suggest that the relationship between social networking site use and mental health is shaped by a complex interaction mechanism, which is influenced and regulated by specific network environments, personal characteristics, and emotions.

The controversy over the relationship between social networking site use and mental health may stem from the failure to clearly distinguish between common social networking site use and problematic social networking site use (PSNSU) (Brand et al., 2016). PSNSU is the excessive use of social networks over a long period with high frequency and intensity, thus damaging the individual’s mental health and physiological functions (Jiang et al., 2016). One survey reported that 17.9% of college students experienced PSNSU (Jolliff et al., 2020). PSNSU was found to be associated with lower happiness (Wang et al., 2019), decreased sleep quality (Yang et al., 2018), and depression (Burnell et al., 2019). Adolescents who frequently use social media are at a higher risk of major depression and suicidal behavior (Twenge et al., 2018). Data from a PSNSU survey of adolescents (*N* = 154,981) in 29 countries showed that PSNSU was related to negative mood and poor life satisfaction (Boer et al., 2020).

### Loneliness and PSNSU

Researchers have generally focused on the factors and mechanisms influencing PSNSU. For example, personality traits, depression, and social anxiety are associated with PSNSU (Acopio and Bance, 2016; Lee et al., 2014). Over the past two decades, the effect of loneliness on PSNSU has attracted widespread interest from researchers. Loneliness is defined as an unpleasant subjective feeling that arises when the quality of one’s actual social relations is not as good as desired and a negative emotional experience resulting from a lack of social interaction skills (Peplau and Perlman, 1982). Yu et al.’s (2016) study found that loneliness is a common feeling among Chinese college students. Owing to their physiological and psychological development, college students are particularly prone to experiencing common and profound loneliness (Chatterjee, 2018). Because college is a period when young people develop their self-identity, students are particularly sensitive to peer interaction and rejection (Orben et al., 2020) and are more likely to feel lonely (Lee et al., 2018). College students with high loneliness experience discomfort in daily interpersonal interactions and self-disclosure and prefer to expand interpersonal relationships through social networks to reduce such discomfort (Hutchins et al., 2021). Compared to their peers, they tend to use social media more intensively (Phu and Gow, 2019) and are at a higher risk of developing an addiction (Biolcati et al., 2018; Rajesh and Rangaiah, 2020).

People experiencing loneliness are often unable to maintain harmonious offline interpersonal relationships, and the asynchronous and relatively secretive nature of social networks makes them more likely to compensate for unmet needs of belonging by seeking satisfaction through social networks (Sharifpoor et al., 2017). In addition, instant, unimpeded access to peers via social networks may increase an individual’s level of interpersonal dependence on such platforms, which, in the long run, may lead to unhealthy dependence on social networks (Davis, 2013). Researchers found that individuals high in loneliness tend to spend more time using mobile social networks and use them more frequently than those low in loneliness (Błachnio et al., 2016). Thus, lonely individuals are more inclined to seek social satisfaction through the Internet, pay more attention to the attitude of others, and more eager to stay in a virtual social life (Käll et al., 2020), which can lead to negative outcomes such as compulsive or excessive online time, unsatisfactory offline interpersonal relationships, and poor academic performance (Masi et al., 2011). According to social compensation theory (Tice, 1993) and social augmentation theory (Valkenburg and Peter, 2009), in the process of people using social media, socially weak or restricted individuals (e.g., lonely individuals) may try to compensate for their shortcomings online. Online networking opportunities may be sought to expand their limited offline social worlds. Concurrently, online social activities provide a sense of belonging, friendship, and communication (Shapira et al., 2000), which may further exacerbate an individual’s lack of offline social connection or other problems, making compensation more important than satisfaction (Brand et al., 2016). Studies have found that loneliness can predict PSNSU (Aalbers et al., 2019; Zhang et al., 2020).

From this perspective, loneliness may predate PSNSU and be one of its causes (Błachnio et al., 2016; Li et al., 2018). However, the researchers believe that the mechanism by which loneliness influences PSNSU is influenced by other variables, and further research is required to increase our understanding (Moretta and Buodo, 2020). One study on the use of social networking sites by young people in the United States found a small negative correlation between loneliness and social networking site use (Pittman and Reich, 2016). In two samples of college students, Skues et al. (2017) found that lonely people may tend to have more Facebook friends and compensate for the lack of offline support by using social media. One cross-hysteresis analysis showed no significant relationship between loneliness and the amount of time individuals spent on Facebook (Wohn and LaRose, 2014). Another study also revealed no statistically significant relation between loneliness and Facebook addiction among high school students (Karakose et al., 2016).

In summary, the prevailing view seems to be that loneliness in young people is positively correlated with PSNSU; however, there may also be a more complex relationship between loneliness and social network use (Nowland et al., 2018). One possible explanation is that most research on the relationship between loneliness and PSNSU is cross-sectional, revealing correlations between variables but not showing causation. Therefore, this study investigated the relationship between loneliness and PSNSU at two different time points. Accordingly, this study proposed Hypothesis 1: loneliness has a significant direct effect on PSNSU among Chinese college students.

### The mediating effect of FoMO

Previous studies have shown that fear of missing out (FoMO) has a significant predictive effect on the PSNSU of college students (Blackwell et al., 2017). Przybylski et al. (2013) described FOMO as ‘a general concern that others may be having a beneficial experience of their own absence’ and manifests as a desire to stay continuously connected to what others are doing’ (p. 1,841). FoMO results from the situational or chronic lack of fulfillment of psychological needs, particularly the lack of stable interpersonal relationships (Przybylski et al., 2013). Loneliness is a subjective negative emotional response to this lack of stable relationships. Self-determination theory states that individuals need to belong to a group and have relationships with others (Tandon et al., 2021). Therefore, when individuals feel out of sync with or miss information from their peers, their sense of belonging may be negatively affected, which could create feelings of anxiety that are reflected in their close and continuous attention to what others are doing. The asynchronous, readily available, and modifiable nature of information on networking sites can undoubtedly satisfy individuals’ sense of belonging (Chai et al., 2018) and relationship needs (Ellison et al., 2014). Thus, individuals with FoMO have a strong desire to keep track of others’ activities, which often leads to problematic social media use behaviors (Chai et al., 2018; Opsenica Kosti’c et al., 2022). Studies have confirmed that People experiencing negative emotions are more likely to engage in PSNSU due to high levels of FoMO (Li et al., 2020a). Therefore, the more the individual experiences feelings of loneliness, the higher the levels of FoMO, which ultimately leads to the overuse of social networking sites. Accordingly, this study proposed Hypothesis 2: FoMO play mediating roles in the influence of loneliness on PSNSU among college students.

Although FoMO may be one factor that can explain why some lonely people develop PSNSU (Dhir et al., 2018), most studies have used the total FoMO score in their analysis (Fioravanti et al., 2021). Recently, however, researchers have found that FoMO may be a more complex structure than a single phenomenon, including trait-FoMO and state-FoMO. Triat-FoMO and state-FoMO are both closely related and distinctly different. Trait-FoMO refers to general anxiety about missing out on something and represents a relatively stable personality trait; state-FoMO refers to the anxiety caused by not keeping up with other people’s updates and interacting with others, which is a kind of unstable cognitive bias (Röttinger et al., 2021). The interaction of the person-affect-cognition-execution (I-PACE) model explains how FoMO contributes to the development of social media addiction (e.g., PSNSU) as a risk factor, suggesting that PSNSU is the result of an interaction between a personality predisposition (e.g., trait-FoMO), cognitive bias (e.g., caused by state-FOMO) and decreased executive ability as a stable personality predisposition (Brand et al., 2019; Starcke et al., 2018).

Some empirical studies have found trait-FoMO, and state-FoMO are closely related to and significantly different from PSNSU (Mao et al., 2023; Hussain et al., 2024): First, trait- and state-FoMO were significantly positively correlated with PSNSU (Wegmann et al., 2021; Hussain et al., 2024). Second, trait-FoMO is significantly associated with PSNSU through the mediation of state-FoMO (Balta et al., 2020). Finally, state-FoMO as a maladaptive cognition could be the most central proximal cause of PSNSU (Wegmann et al., 2017; Li et al., 2024). In a study of Chinese college students, Shi et al. (2022) also found that trait-FOMO could not only directly predict college students’ smartphone addiction but also indirectly predict it by the mediating effect of state-FoMO. These results seem to point to the possibility that relatively stable personality traits (i.e., trait-FoMO) can predict specific cognitions in an online environment (i.e., state-FoMO), with state-FoMO acting as an intermediary between trait-FoMO and PSNSU. Accordingly, this study proposed Hypothesis 3: state-FoMO acts as an intermediary between trait-FoMO and PSNSU.

### Present study

Previous studies have not distinguished whether trait-FoMO or state-FoMO predicted PSNSU, let alone discuss the mechanism of action of these two types of FoMO. Therefore, this study aims to investigate whether loneliness can predict PSNSU and whether trait- and state-FoMO mediate the relationship between the two. Especially in an environment in which social networks continue to evolve and upgrade, the correct distinction between trait- and state-FoMO is meaningful to further explore the mechanism of interaction between loneliness and PSNSU. The interaction of the person-affect-cognition-execution (I-PACE) proposed by Brand et al. (2016) has provided valuable insight to this study. Brand et al. (2019) suggest that in the early stages of behavioral addiction, negative emotions (e.g., loneliness) may be perceived as internal triggers, leading to an interaction of personality predisposition (e.g., trait-FoMO) and cognitive biases (e.g., caused by state-FoMO) that leads to relevant experiences of satisfaction and compensation, and in turn to more highly engaged social media.

**Figure 1:**
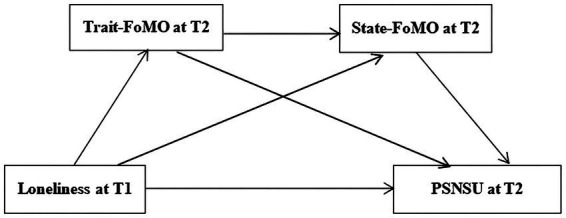
The proposed multiple mediation model. FOMO, fear of missing out; PSNSU, problematic social networking site use.

Given the above, based on the I-PACE model, this study constructed a multi-mediating model with loneliness as the independent variable, PSNSU as the dependent variable, and trait-FoMO and state-FoMO as the mediating variables (see Figure 1). This study not only examined the causal relationship between loneliness and PSNSU but also revealed the different roles of the two subtypes of miss anxiety, trait FOMO and state FOMO, in the relationship between loneliness and PSNSU in college students.

## Materials and methods

### Participants

According to G*Power 3.1.9.7 software, to obtain a medium effect size (*f*^2^ = 0.15) and sufficient statistical testing power (1-*β* = 0.95, *α* = 0.05), a sample size of 119 was required (Faul et al., 2007). Data were collected in two rounds from students from two universities in Hubei Province in China. Loneliness, PSNSU, and demographic information (gender age, and grade) were measured in the 434 students who engaged in the first data collection in November 2022 (T1). The second round of data collection was conducted in November 2023 to measure FoMO and PSNSU. Overall, 17 students failed to participate in the second test because of transfer, leave, and other reasons, resulting in 417 valid questionnaires, with a loss rate of 3.92%. Among the 417 participants, 188 were male students (45.08%), and 229 were female students (54.92%). The ages of the participants ranged from 17 to 23 years old, with an average age of 19.87 years (*SD* = 1.05 years). The participants consisted of 143(34.3%) in Grade One, 158 (37.9%) in Grade Two, and 116 (27.8%) in Grade Three. The independent sample *t-*test was conducted for the lost participants (*n* = 17) and the effective participants (*n* = 417). The results indicated no statistically significant differences in the loneliness scores (*t* = 0.33) and PSNSU (*t* = 0.45) between the two groups (*p* > 0.05), suggesting that the sample loss was random.

### Measures

#### UCLA Loneliness Scale

The UCLA Loneliness Scale (ULS-8) (Hays and DiMatteo, 1987) was used to assess loneliness. The scale consists of eight items (e.g., “*I have people around me, but no one cares about me*”). Items are measured on a 4-point Likert scale (1 = *never* to 4 = *all the time*), for example, “I have people around me, but no one cares about me.” Higher scores indicate that the individual feels lonelier. The Cronbach’s *α* coefficient was 0.84 in this study.

#### FoMO Scale

The Fear of Missing Out (FoMO) Scale (Wegmann et al., 2017) comprises 12 items, including two dimensions: trait-FoMO (five items, such as *I’m afraid that other people will have more novel experiences than I do*) and state-FoMO (7 items, such as *I kept surfing the Internet, hoping not to miss anything important*). Items are measured on a 5-point Likert scale (1 = *strongly disagree* to 5 = *strongly agree*). A higher score indicates higher levels of FoMO. The Cronbach’s *α* of each dimension subscale in this study were 0.76 and 0.82, respectively.

#### Social Networking Site Addiction Scale

The Social Networking Site Addiction Scale (Chen et al., 2018) is a revised version of the Facebook Addiction Scale (Koc and Gulyagci, 2013) and was used to assess social networking site addiction. It consists of eight items (e.g., “*Every morning when I wake up, the first thing I think about is logging on to social networking sites*”). Items are measured on a 5-point Likert scale (1 = *never* to 4 = *all the time*). Higher scores indicate more severe problematic social networking site use. Cronbach’s *α* for the pre- and post-measurement scales were 0.87 (T1) and 0.84 (T2), respectively, in this study.

### Procedure

Ethical approval was obtained from the Ethics Committee of Hubei Engineering University. We published recruitment information in the students’ QQ group through the counselors at both universities and distributed questionnaires at the college’s centralized activities. Two waves of data were collected from the participants at two time points (T1 and T2) 1 year apart. Written informed consent was obtained from all participants before the data collection. All participants who participated in the survey understood the purpose and were aware of the principle of confidentiality and voluntariness. Participants were informed that their participation was voluntary and that they could terminate their participation at any time. During school hours, we measured all questionnaires on one school day, and the questionnaires were administered in paper-and-pencil form. The tests and questionnaires were administered in the classroom during class time. The questionnaire took approximately 20 min to complete. Additionally, 6-trained graduate students completed the questionnaire distribution and recycling according to standard procedures. There were 417 valid questionnaires in both formal tests.

### Data analysis

SPSS 26.0 was used for descriptive statistics and correlation analysis. Amos 24.0 was used to test the mediation model proposed. Next, we used Model 6 of the PROCESS macro program in SPSS (Hayes, 2012) to analyze the mediation model proposed. Five-thousand bootstrap samples were drawn from the entire dataset. We used a 95% confidence interval (CI) to determine the significance of the mediation effect. To control for methodological bias arising from the self-report questionnaires, the Harman single-factor test was used to assess common method bias across the variables. The results indicated 14 factors with eigen roots greater than 1, and the first factor explained 20.44% of the variance, which is below the critical value of 40%. The results proved that this study does not suffer from significant common method bias (Podsakoff et al., 2003).

## Results

### Descriptive statistics and correlation analysis

The descriptive statistical analysis and correlation matrix of each research variable are shown in Table 1. Loneliness at T1 was significantly positively correlated with trait-FoMO at T2 (*r* = 0.22, *p* < 0.01), state-FoMO at T2 (*r* = 0.14, *p* < 0.01), PSNSU at T1 (*r* = 0.15, *p* < 0.01) and PSNSU at T2 (*r* = 0.13, *p* < 0.05). Trait- and state-FoMO at T2 were significantly positively correlated with PSNSU at T2 (*r* = 0.36, 0.32, *p* < 0.01, respectively). Trait-FoMO at T2 was positively correlated with state-FoMO at T2 (*r* = 0.20, *p* < 0.01). Gender was correlated with trait-FoMO at T2 (*r* = 0.22, *p* < 0.01), state-FoMO at T2 (*r* = 0.19, *p* < 0.01), PSNSU at T1 (*r* = 0.16, *p* < 0.01) and PSNSU at T2 (*r* = 0.15, *p* < 0.01). These results indicate close correlations among the main variables, suggesting the need to test for mediating effects in combination.

**Table 1:** Means, SD, and correlation matrix.

Variables	1	2	3	4	5	6	7
1. Gender	1						
2. Age	−0.17**	1					
3. Loneliness (T1)	0.02	−0.02	1				
4. Trait-FoMO (T2)	0.22**	0.07	0.22**	1			
5. State-FoMO (T2)	0.19**	−0.02	0.14**	0.20**	1		
6. PSNSU (T1)	0.16**	−0.02	0.15**	0.13*	0.21**	1	
7. PSNSU (T2)	0.15**	0.06	0.13*	0.36**	0.32**	0.45**	1
*M*		19.87	1.92	2.22	2.17	2.50	2.53
*SD*		1.05	0.43	0.71	0.62	0.57	0.68

**p < 0.01, *p < 0.05.

FOMO, fear of missing out; PSNSU, problematic social networking site use.

### Testing for multiple mediating effects

**Figure 2:**
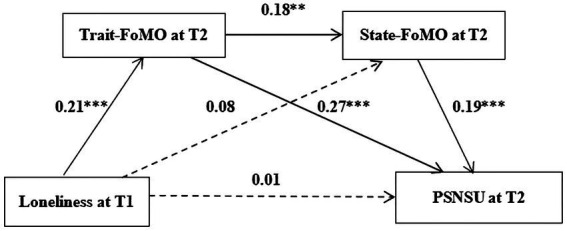
The multiple mediating effects of trait-FoMO and state-FoMO. The significant path is solid line and the non-significant path is a dashed line. ***p < 0.001, **p < 0.01. For brevity, the control variables are not shown.

Controlling for gender and PSNSU at T1, multiple mediation model tests were conducted with loneliness at T1 as the independent variable, trait-FoMO at T2 and state-FoMO at T2 as the mediating variables, and PSNSU at T2 as the dependent variable (see Figure 2). The results showed that the model fit well: *χ*^2^/*df* = 1.25, CFI = 0.998, TLI = 0.983, RMSEA = 0.027, and SRMR = 0.018.

First, the predictive effect of loneliness at T1 on PSNSU at T2 was investigated. The results showed that loneliness at T1 significantly predicted PSNSU at T2 (*β* = 0.09, *t* = 2.00, *p* < 0 0.05). The results showed Hypothesis 1 was supported. However, as shown in Table 2, when considering trait-and state-FoMO at T2 as mediators, loneliness at T1 did not significantly predict PSNSU at T2 (*β* = 0.01, *p >* 0.05) and state-FoMO at T2 (*β* = 0.08, *p >* 0.05), but significantly positively predicted trait-FoMO at T2 (*β* = 0.21, *p* < 0.001). In addition, trait-FoMO at T2 significantly positively predicted state-FoMO at T2 and PSNSU at T2 (*β* = 0.18, *p* < 0.01 and *β* = 0.27, *p* < 0.001, respectively), and state-FoMO significantly positively predicted PSNSU at T2 (*β* = 0.19, *p* < 0.001).

**Table 2:** Testing for the multiple mediating effects.

Regression equation (*N* = 417)		Fitting indicators			Coefficient significance	
Outcomes	Predictors	*R*	*R2*	*F*	*β*	*t*
Trait-FoMO (T2)		0.33	0.11	14.33***		
	Gender				0.44	3.83***
	PSNSU (T1)				0.08	1.61
	Loneliness (T1)				0.21	4.60***
State-FoMO (T2)		0.33	0.11	10.38***		
	Gender				0.32	2.38*
	PSNSU (T1)				0.18	3.15**
	Loneliness (T1)				0.08	1.55
	Trait-FoMO (T2)				0.18	3.04**
PSNSU (T2)		0.57	0.33	33.36***		
	Gender				−0.01	−0.13
	PSNSU (T1)				0.39	8.26***
	Loneliness (T1)				0.01	0.25
	Trait-FoMO (T2)				0.27	5.21***
	State-FoMO (T2)				0.19	4.30***

***p < 0.001, **p < 0.01, *p < 0.05.

FOMO, fear of missing out; PSNSU, problematic social networking site use.

To further investigate the relationship between loneliness and PSNSU, 5,000 bootstrap samples were drawn from the entire dataset. As shown in Table 3, the 95% CI of the direct path coefficient of the effect of loneliness on PSNSU is [−0.08, 0.09], and the 95% CI of the mediating effect of state-FoMO is [−0.004, 0.04], both which include zero. Trait-FoMO and state-FoMO fully mediated the relationship between loneliness and PSNSU, and the total mediating effect value was 0.08. The mediating effect consists of two pathways. The first pathway was loneliness → trait-FoMO → PSNSU with an effect size of 0.06 and a 95% CI [0.03, 0.09]. Therefore, Hypothesis 2 is partly supported. The second pathway was loneliness → trait-FoMO → state-FoMO → PSNSU with an effect size of 0.01 and a 95% CI [0.002, 0.02]. Obviously, the chain mediating effect of ‘trait-FoMO to state-FoMO’ was significant, supporting Hypothesis 3. It can be concluded that the influence of loneliness on PSNSU of college students 1 year later was realized through the single mediating effect of trait FoMO and the chain mediating effect of trait-FoMO and state-FoMO.

**Table 3:** The estimates of the total, direct, and indirect effects of the model.

Effect		Boot SE	Boot LLCI	Boot ULCI
Total effect	0.09	0.05	0.002	0.18
Direct effect	0.01	0.05	−0.08	0.09
Total indirect effect	0.08	0.02	0.04	0.12
Loneliness (T1) → Trait-FoMO (T2) → PSNSU (T2)	0.06	0.02	0.03	0.09
Loneliness (T1) → State-FoMO (T2) → PSNSU (T2)	0.01	0.01	−0.004	0.04
Loneliness (T1) → Trait-FoMO (T2) → State-FoMO (T2) → PSNSU (T2)	0.01	0.004	0.002	0.02

FOMO, fear of missing out; PSNSU, problematic social networking site use.

## Discussion

### Loneliness and PSNSU

Our results revealed that loneliness had a significant predictive effect on PSNSU. However, after adding the mediating variables of FoMO, the direct effect of loneliness on PSNSU was neutralized. This result is consistent with Beyens et al. (2021), who pointed out that the effect of emotional, psychological indicators on PSNSU may be influenced by other variables, showing a more complex mechanism.

The relationship between loneliness and PSNSU has received increasing attention from researchers over the past two decades. Błachnio et al. (2016) found the amount of time and frequency of using mobile social media was much higher in individuals who were lonelier than those who were less lonely. Some studies have found that loneliness was a predictor of PSNSU (Aalbers et al., 2019; Reissmann et al., 2018). Cross-lag panel analysis of a longitudinal study showed that loneliness at T1 was positively correlated with social media use at T2 (Zhang et al., 2020). Thus, loneliness is a significant predictor of PSNSU (Lee et al., 2017; Shettar et al., 2017; Li et al., 2018). However, some studies contradict these findings, showing a weak or no correlation between loneliness and PSNSU (Karakose et al., 2016; Kircaburun et al., 2020). Two longitudinal studies revealed that loneliness did not predict PSNSU (Kross et al., 2013). In another longitudinal study that included two time points (Yang et al., 2019), loneliness at T1 did not predict PSNSU at T2, and the relationship between loneliness and PSNSU may have been influenced by mediating variables such as social comparison.

Nevertheless, loneliness seems to be a risk factor for PSNSU, but perhaps it does not directly trigger PSNSU (O’Day and Heimberg, 2021). Instead, loneliness indirectly influences PSNSU through different mediating variables. When investigating the relationship between loneliness and PSNSU without controlling for other variables in the analysis, most previous studies have reported a moderately positive association (Moretta and Buodo, 2020). However, after controlling for factors such as social support and depression (Ozsaker et al., 2015), family function and self-esteem (Shi et al., 2017), lack of face-to-face social time (Costa et al., 2019) and belonging to the same online community (Savolainen et al., 2020), the relationship between loneliness and PSNSU is weaker. A longitudinal study found a positive correlation between loneliness and problematic Internet use, but other relevant variables, such as anxiety, might affect the relationship (Moretta and Buodo, 2020). Thus, loneliness does not directly trigger PSNSU in college students; however, when they try to relieve the psychological discomfort of loneliness, they may frequently use social networking sites to avoid losses, such as loss of information, reputation, relatedness, or popularity (Alutaybi et al., 2019), leading to the development of PSNSU.

### The mediation analysis

Although previous research has suggested a link between FoMO and PSUSN (Dou et al., 2023), this study confirms how FoMO operates at both trait- and state-levels to promote PSNSU in college students. Specifically, this study found different mediating pathways through which FoMO affects the connection between loneliness and PSNSU. The mediating role of trait-FoMO was significant, whereas the mediating role of state-FoMO was not significant.

Similar to previous studies, this study revealed that loneliness is significantly correlated with trait-FoMO but not state-FoMO (Mao et al., 2023; Fumagalli et al., 2021). Several studies have found that trait-FoMO is closely related to negative emotions such as depression and loneliness, which is the result of unsatisfied basic psychological needs (especially the need for belonging) (Yin et al., 2021). Researchers found that loneliness and FoMO share the same topological deviations in the resting-state EEG brain network (Yin et al., 2023). As a relatively stable personality trait, FoMO has a neural basis and is more likely to be closely related to loneliness (Yin et al., 2023). Röttinger et al. (2021) confirmed that mental health status was unrelated to state-FOMO, whereas it was negatively correlated with trait-FoMO, which in turn affected problematic Internet use. Mao et al. (2023) also revealed that loneliness was more closely related to trait-FoMO. Mao et al. (2024) found that the risk of social media addiction in trait-FoMO group was greater than that in the state-FoMO group through latent profile analysis. This may be because trait-FoMO is a more stable personality tendency (Wegmann et al., 2017), which amplifies the impact of external factors on individuals, resulting in persistent anxiety and an increased risk of problematic social media use (Balta et al., 2020). Thus, as the findings of this study demonstrated, loneliness increases the risk of PSNSU through trait-FoMO rather than state-FOMO.

The current study also revealed that trait FoMO, as a stable personality predisposition, predicts state-FoMO, which is a significant difference between trait-FoMO and state-FoMO (Balta et al., 2020). Unlike the stable personality trait represented by general trait-FoMO, state-FoMO represents the specific unstable and negative cognition developed during online activities (Xiao and Liu, 2019) and is more closely related to PSNSU (Li et al., 2020b; Montag et al., 2023). Another study found that among social network users, trait-FoMO was found to mediate the association between mental health and Internet use disorders, whereas state-FoMO mediated the association between trait-FoMO and Internet use disorders (Röttinger et al., 2021). This suggests that online-specific state-FoMO in social networking site use is caused by a general fear of disconnection (general trait-FoMO) rather than directly by negative emotions such as loneliness.

Moreover, according to the I-PACE model, a core characteristic predisposing individuals to PSNSU may be the initial trigger to Internet-related maladaptive cognitions. These cognitions often lead to short-term positive experiences and satisfaction online. However, as usage time increases, the initial satisfaction diminishes, and the compensatory effect increases. During social media use, individuals may attempt to compensate for deficiencies in real life (such as poor interpersonal relationships and negative emotional experiences). This compensatory behavior can also heighten the desire for stimulating situations and ultimately strengthen PSNSU (Brand et al., 2016, 2019). General trait-FoMO, known as the widespread fear of disconnection, is also included in the I-PACE model as a related susceptibility personality trait (Brand et al., 2016). Trait-FoMO causes changes in the individual’s state characteristics, leading to excessive demand for Internet use and causing PSNSU. Montag et al. (2023) and Hussain et al. (2024) found that both trait-FoMO and state-FoMO were significantly correlated with PSNSU; however, state-FoMO was the proximal factor more closely associated with PSNSU. Based on the above analysis, the relationship between loneliness and PSNSU involved the following process: loneliness → trait-FoMO → state-FoMO → PSNSU.

The results of this study revealed how loneliness influences PSNSU through both trait-FoMO and state-FoMO. Different from previous studies that took FoMO as a unified construct (Zhang et al., 2021), this study distinguished between trait-FoMO and state-FoMO, revealing that trait-FoMO poses a higher risk of addiction and will indirectly affect PSUSN through state-FoMO. This study also responded well to the call of Elhai et al. (2020) to distinguish between trait-FoMO and state-FoMO, and their suggestion that more research is necessary to explore their different roles.

### Limitations and implications

Based on the I-PACE model, this study aimed to examine the relationship between Chinese college students’ loneliness and PSNSU, along with its underlying mechanisms, helping to understand the differences in how the two types of FoMO affect the link between loneliness and PSNSU. However, the study had some limitations. First, the study was conducted at two time points 1 year apart. Although questionnaire measurements are valid for investigating the causal relationships between variables, more time points are needed to obtain a more comprehensive understanding of the underlying mechanisms of loneliness affecting PSNSU. Second, the research data were all from the self-rated reports of the participants, which may be affected by their subjective defenses and social expectations. This method of data collection could lead to errors or exaggerated correlations due to common methodological biases. Future studies could incorporate multiple perspectives, including parents’, peers’, and teachers’ evaluations, to obtain a more comprehensive understanding of the variables. Third, this study revealed the different mechanisms of trait- and state-FoMO on PSNSU. However, the theoretical and empirical differences between them need to be further investigated (Hussain et al., 2024; Li et al., 2024). Future research should incorporate other research methods, such as qualitative or experimental approaches, to collect data from various perspectives and deepen the understanding of the different mechanisms of trait- and state-FoMO on PSNSU in college students. Lastly, the sample was recruited from two universities in a province in central China, so caution should be required when interpreting the findings within different regions or outside of China. In addition, we encourage future studies to investigate other representative samples (e.g., adolescents and older adults).

Despite these limitations, this study deepened and expanded our understanding of the causal relationship between loneliness and PSNSU and trait- and state-FoMO as underlying mechanisms in the relationship. Loneliness and PSNSU were positively correlated at both time points (i.e., at the cross-sectional level). However, there was no direct longitudinal correlation between them after the inclusion of FoMO as a mediating variable, indicating that loneliness has a more complex mechanism of action on PSNSU. Additionally, the finding highlighted the importance of examining mediating variables that may affect this relationship. Structural equation models documented the complex associations between loneliness and PSNSU mediated by two dimensions of FoMO. Consistent with the research results of Wegmann et al. (2017), FoMO is a complex multi-dimensional psychological structure. This study provided robust evidence for differentiating the mechanisms of differential action of trait-and state-FoMO on PSNSU.

The results of this study have practical implications for PSNSU and FoMO intervention in college students. First, considering that loneliness and trait-FoMO may be remote causes of PSNSU, making individuals prone to Internet-related maladaptive cognitions, it is necessary to increase offline interpersonal communication activities, improve self-evaluation, and meet the need for belonging. Mao et al.’s (2023) study confirmed that interpersonal satisfaction is highly correlated with low trait-FoMO. Second, considering that state-FoMO is a proximal cause of PSNSU, college students can potentially prevent PSNSU by participating in offline activities actively, such as sports and leisure activities (Brailovskaia et al., 2018; Tomczyk and Selmanagic-Lizde, 2018). In addition, college students should place less importance on receiving timely responses to their posts and eliminate the interference of external factors by deleting or shutting down some communication software (Hunt et al., 2018). Researchers should aim to change the functional design of social media to reduce users’ levels of state-FoMO (Alutaybi et al., 2020). Third, considering that FoMO represents an obstruction to individual self-emotional and cognitive regulation (Przybylski et al., 2013), cognitive behavioral methods can be used to treat PSNSU. This helps reduce individuals’ immediate attention to and responses to asynchronous information on social networks. Moreover, cognitive behavioral therapy is effective in treating problematic Internet use and FoMO (Stevens et al., 2019).

## Conclusion

Unlike previous studies, which took FoMO as a unified construct (Zhang et al., 2021), this study compared the different mechanisms by which the two types of FoMO affect PSNSU, demonstrating that trait-FoMO and state-FoMO fully mediate the relationship between loneliness and PSNSU. This implies that the direct effect of loneliness on PSNSU is neutralized when trait-FoMO and state-FoMO are introduced as mediating variables. Loneliness affected PSNSU through the single mediating effect of trait-FoMO and the chain mediating effect of trait-FoMO and state-FoMO, confirming the research results based on the I-PACE model (Brand et al., 2019). This indicates that college students who engage excessively in WeChat, Weibo, QQ, and Xiaohongshu because of negative emotions should be helped to realize that a general fear of social disconnection represented by trait-FoMO significantly contributes to PSNSU. They should be encouraged to develop greater initiative in face-to-face interpersonal communication to address the root cause and ultimately reduce the risk of PSNSU. Meanwhile, considering that state-FoMO is a proximal cause of PSNSU, the most effective way to reduce the impact of FoMO on our lives is to live life without being reliant on technological devices. College students should be able to disengage from their phones and laptops at any time.

## Data Availability

The raw data supporting the conclusions of this article will be made available by the authors, without undue reservation.
